# Growth, Viability, and Death of Planktonic and Biofilm *Sphingomonas desiccabilis* in Simulated Martian Brines

**DOI:** 10.1089/ast.2018.1840

**Published:** 2018-12-29

**Authors:** Adam H. Stevens, Delma Childers, Mark Fox-Powell, Natasha Nicholson, Elisha Jhoti, Charles S. Cockell

**Affiliations:** ^1^ UK Centre for Astrobiology, School of Physics and Astronomy, University of Edinburgh, Edinburgh, United Kingdom.; ^2^ Aberdeen Fungal Group, Institute of Medical Sciences, MRC Centre for Medical Mycology at the University of Aberdeen, Aberdeen, United Kingdom.; ^3^ School of Earth and Environmental Sciences, University of St. Andrews, St. Andrews, United Kingdom.

**Keywords:** Mars, Brines, Extremophile, Halotolerance, Desiccation

## Abstract

Aqueous solutions on Mars are theorized to contain very different ion compositions than those on Earth. To determine the effect of such solutions on typical environmental micro-organisms, which could be released from robotic spacecraft or human exploration activity, we investigated the resistance of *Sphingomonas desiccabilis* to brines that simulate the composition of martian aqueous environments. *S. desiccabilis* is a desiccation-resistant, biofilm-forming microbe found in desert crusts. The viability of cells in both planktonic and biofilm forms was measured after exposure to simulated martian brines. Planktonic cells showed a loss of viability over the course of several hours in almost all of the seven brines tested. Biofilms conferred greater resistance to all the brines, including those with low water activity and pH, but even cells in biofilms showed a complete loss of viability in <6 h in the harsher brines and in <2 days in the less harsh brines. One brine, however, allowed the microbes to maintain viability over several days, despite having a water activity and pH lower and ionic strength higher than brines that reduced viability over the same timescales, suggesting important ion-specific effects. These data show that biofilm-forming cells have a greater capacity to resist martian aqueous extremes, but that evaporative or deliquescent brines are likely to be destructive to many organisms over relatively short timescales, with implications for the habitability of Mars and for micro-organisms dispersed by robotic or human explorers.

## 1. Introduction

The search for habitable environments in the universe centers on the availability of liquid water, and aqueous environments are now known to exist on a number of planetary bodies, including Mars, Europa, Enceladus, and Titan (Fortes, 2000; Kivelson *et al.*, 2000; Mellon and Phillips, 2001; Squyres *et al.*, 2004; Postberg *et al.*, 2011). Many of these aqueous environments contain combinations of anions that are rare on Earth. For example, the high concentrations of sulfate and other divalent ions in martian brines, a consequence of the specific geological history of that planet, have consequences for habitability, making ionic strength an important determinant of whether the brines will support life (Fox-Powell *et al.*, 2016).

The presence of brines at or near the surface of Mars has been proposed since early exploration missions (Brass, 1980; Zent and Fanale, 1986). Continued discovery of evidence for past hydrological systems (Knoll *et al.*, 2005) and even signs of present hydrological activity (Hecht *et al.*, 2009; Ojha *et al.*, 2015) suggest that Mars hosted or perhaps still hosts aqueous environments, but that these environments would have been drastically different than typical aqueous environments on Earth. Proposed martian brines require high levels of dissolved salts to remain liquid under martian surface conditions and are typically rich in ions not typically found in terrestrial aqueous environments, including sulfate, iron, and perchlorate (Chevrier and Altheide, 2008; Marion *et al.*, 2010), and would likely have been highly acidic (Squyres and Knoll, 2005). Recent observations and measurements suggest that there is still aqueous activity at the martian surface, perhaps flowing from a subsurface aquifer or deliquescing from the atmosphere due to the presence of salts in the surface regolith (Heinz *et al.*, 2016; Bhardwaj *et al.*, 2017). These observations imply an aqueous environment on the surface of Mars that is transiently wetted and desiccated at unknown timescales (Martin-Torres *et al.*, 2015). Martian surface conditions coupled with the inferred composition of martian brines mean that these aqueous environments will be unlike any on Earth, making their habitability unknown.

An environment with repeated cycles of matric (drying) and osmotic (saline) water stress presents a harsh setting for any potential inhabitants, even before the additional extreme conditions at Mars' surface. However, there are many terrestrial organisms that are resistant to matric/osmotic stresses. Laboratory studies have shown that spores are resistant to some Mars-relevant deliquescent brine environments (Nuding *et al.*, 2017), but the exotic sulfate- and perchlorate salts implied to exist on Mars (Tosca *et al.*, 2011) represent conditions that have not been extensively tested using potentially resistant terrestrial micro-organisms. *Sphingomonas desiccabilis* is a Gram-negative, nonmotile, biofilm-forming bacterium extracted from an arid soil of the Colorado Plateau that is desiccation resistant and moderately osmotolerant (Reddy and Garcia-Pichel, 2007). *Sphingomonas* species have also been identified in spacecraft assembly clean rooms (Moissl *et al.*, 2007). We investigated the survival of *S. desiccabilis* in response to simulated martian brines as an example of an organism that might share features with a putative martian soil inhabitant.

One of the mechanisms used to protect against matric and osmotic stress is the excretion of extracellular polymeric substances (EPSs) and the formation of biofilms (Zhang *et al.*, 2014, 2015). Biofilms are important in understanding microbial survival and growth in extremes because they offer resistance against a number of extreme conditions, including bactericidal chemicals (Luppens *et al.*, 2002), rapid changes in temperature and pH (Koerdt *et al.*, 2010), and ultraviolet radiation (Niemira and Solomon, 2005). These physical stresses are all associated with martian surface conditions.

The investigation of whether biofilms confer resistance to extraterrestrial extremes, particularly brines, has implications for planetary protection and how they might influence microbial survival in extreme extraterrestrial environments. In particular, the definition of “special regions” (Rettberg *et al.*, 2016) and whether terrestrial organisms accidently introduced into extraterrestrial brines by spacecraft could persist in these regions require an understanding of their potential habitability with respect to terrestrial organisms. These implications are relevant for Mars but also for the icy moons in the outer solar system, which are thought to have high levels of dissolved salts in their liquid water interiors (Hand and Carlson, 2015). Understanding how different microbes are able to survive in these environments is important for understanding their habitability.

## 2. Materials and Methods

### 2.1. Strains and growth conditions

**FIG. 1.**
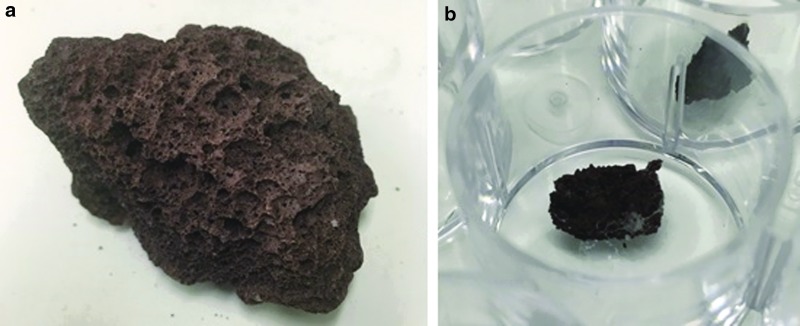
Basaltic scoria was used as a growth substrate **(a)** larger ∼2 cm piece and **(b)** broken down to ∼5 mm piece for use in well plates.

This study examined viability and biomass in the organism *S. desiccabilis*, a Gram-negative, rod-shaped, chemoheterotrophic, strictly aerobic bacterium originally isolated from a soil crust in the Colorado Plateau, USA (Reddy and Garcia-Pichel, 2007) and obtained from the DSMZ culture collection (Type strain 16792). Overnight cultures of *S. desiccabilis,* grown in R2A medium at 20°C (Reasoner and Geldreich, 1985) in a shaking incubator, were normalized by cell density using OD_600_ measurements. Pellets of planktonic cells were created by centrifuging 1 mL of OD_600_ = 1 culture at 2,400 *g* for 5 min and by removing the supernatant. Biofilms were prepared by inoculating the same overnight cultures onto autoclaved ∼5 mm pieces of Mars-analog regolith substrate at a concentration of 50 μL in 4 mL of R2A medium and grown for 1 week at 20°C. The substrate used was highly vesiculated Icelandic lava (Bar-Be-Quick Barbeque Products, Burnley, UK) with a basaltic composition (Fig. 1). In this study, the substrate was chosen as a generic vesiculated rock material for biofilm formation rather than as a strict geochemical analogue to martian surface material.

After a week of growth, the supernatant was removed from the scoria. The biofilm-covered scoria pieces were then either (a) dried in a laminar flow hood overnight (16 h) at 10% relative humidity (RH) and 28°C, or (b) had their media replaced with phosphate-buffered saline (PBS) to halt growth but prevent desiccation. This allowed us to compare the response of planktonic cells, desiccated biofilms, and hydrated biofilms.

### 2.2. Desiccation tolerance

The desiccation tolerance of *S. desiccabilis* was investigated. Overnight cultures of *S. desiccabilis* were serially diluted and plated onto R2A agar to enumerate the population by colony-forming units (CFUs) before desiccation.

Desiccated cells were prepared on flame-sterilized glass slides in a laminar flow hood with three 20 μL spots of culture solution. The glass slides were dried in a flow hood at 10% RH and 28°C and secured in Petri dishes with Parafilm, and then stored on the bench at room temperature.

Desiccated cell spots were rehydrated with 75 μL of R2A per drop by pipette mixing 25 μL R2A at a time until the slide appeared clear. The 75 μL of rehydrated *S. dessicabilis* was then serially diluted to and plated on R2A agar. CFUs were counted after 2 days.

### 2.3. Synthesis of brines and exposure

The brines were synthesized based on computational reconstructions of putative martian brines by Tosca *et al.* (2011), as used by Fox-Powell *et al.* (2016). They included four types at two stages in their evaporation sequence (a and b, corresponding to early- and late-stage evaporation). Type Ia and Ib are alkaline carbonate-chloride brines, which are analogous to the conditions in Gale Crater ∼3.7 billion years ago (Léveillé *et al.*, 2014) and the fluids that interacted with the Nakhla martian meteorite (Bridges and Schwenzer, 2012). Type IIa and IIb are Mg-SO_4_-Cl-dominated brines, characteristic of widespread Hesperian evaporite deposits such as those at Meridiani Planum (Knoll *et al.*, 2005). Type IIIa and IIIb are similar in composition to type II but contain higher levels of dissolved iron and are extremely acidic.

In addition to the six Mars-analog brines, a magnesium perchlorate solution was included (type IV), given observations of magnesium perchlorate in the martian polar regolith by the Phoenix lander (Hecht *et al.*, 2009), and more recently associated with Recurrent Slope Lineae (RSL) following spectral observations (Ojha *et al.*, 2015). This brine was a solution of magnesium perchlorate with a concentration to give a water activity close to that of brine IIb to investigate any differences caused by the chemical properties of the perchlorate salt. The molar recipes used to produce these brines are shown in Table 1, along with measurements of their physicochemical properties.

**Table 1.** Salts Added to Mix Mars-Analog Brines

*Brine type*	*Ia*	*IIa*	*IIIa*	*Ib*	*IIb*	*IIIb*	*IV*
NaHCO_3_	0.126						
KHCO_3_	0.028	0.041		2.237			
KCl	0.022	0.020	0.075	3.776	1.033	1.142	
MgCl_2_.6H_2_O	0.001	0.056			1.154	3.007	
NaCl		0.154	0.189	1.266	2.265	1.036	
MgSO_4_.7H_2_0		2.068	3.066		2.550		
FeSO_4_.7H_2_0			1.225			2.313	
FeCl_2_.4H_2_O			0.208			0.985	
HCl					0.038	0.113	
Mg(ClO_4_)_2_							3.097
Water activity	0.97	0.92	0.9	0.8	0.63	0.56	0.67
pH	8.2	6.2	1.8	8.3	2	0.6	5.5
Ionic strength, mol/L	0.18	8.5	12.0	5.0	11.6	13.0	9.29

Concentrations are given in mol/L. Adapted from Fox-Powell *et al.* (2016) and originally calculated from Tosca *et al.* (2011), except brine IV, which was designed for this study.

The planktonic cell pellets and biofilm-inoculated scoria were treated with these Mars-analog brines over a time course. Cell pellets were treated with 1 mL of each brine (with PBS as a nonbrine control) and vortexed briefly to mix. Biofilm-coated scoria was covered with 4 mL of each brine.

Transient and repeated brining was tested separately by preparing scoria pieces in the same way as described above but removing the brines from the scoria after an hour. After removing the brine, the biofilm-coated scoria was dried again in a sterile flow hood (at 10% RH and 28°C) until there was no visible surface moisture on the scoria. This brining and drying process was repeated three times.

### 2.4. Viability after brining

After being immersed in the brine, the brined planktonic cells were diluted with 1 mL of water to reduce the fluid density and allow for efficient centrifugation and centrifuged at 9,600 *g* for 5 min. The supernatant was removed, and the cells were resuspended in 1 mL of PBS. The brined scoria was washed twice in PBS, and then suspended in 1 mL of PBS. The suspension was vortexed briefly and sonicated for 2 min to extract cells from the biofilms. While this process will not necessarily have removed all the cells from the biofilms, we assumed the extraction efficiency was consistent for a given amount of vortexing and sonication.

In all three cases, cell suspensions were plated onto nutrient agar plates in serial dilution, and the CFUs were quantified after 2 days of growth at 20°C to give a measure of viability after immersion after a given amount of time in the different brines. Results were collected for each brine for planktonic cells and both dried and hydrated biofilms in biological triplicate and systematic replicate at different time points. In the latter cases CFU counts were normalized to the mass of scoria on which the biofilms were grown.

### 2.5. Determination of biomass

We performed a crystal violet (CV) staining to investigate how the brines affect the overall biomass of the biofilms. Biofilms were grown by using the same method as described above and stained by using a protocol adapted from the work of Childers *et al.* (2013). The biofilm-coated scoria was incubated in different brines, or PBS as a control, for 24 h, and then washed three times in PBS. Each scoria piece was then stained with 0.4% CV solution for 15 min at room temperature, washed three times with PBS and destained for 15 min in 1 mL of 33% acetic acid solution. The optical density of the destain solution was used to quantify the total biomass of biofilm left on each piece of scoria after brining, with the PBS incubation forming the “no brine” control. The OD_570_ was measured by spectrophotometer with 33% acetic acid as the blank.

## 3. Results

### 3.1. Desiccation tolerance

**FIG. 2.**
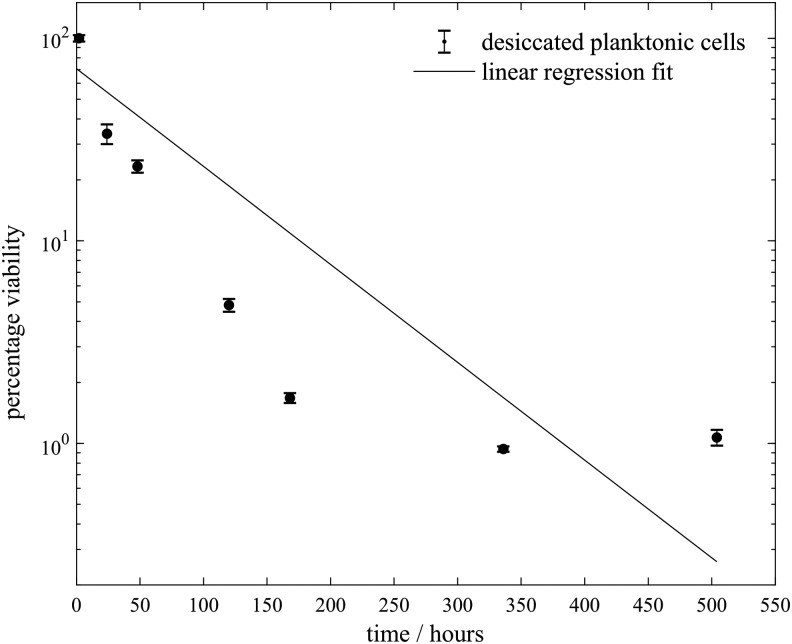
Viability of *Sphingomonas desiccabilis* cells in planktonic form after desiccation for extended periods of time. A trend line is plotted as a linear regression of all collected data.

To confirm the desiccation resistance inferred by Reddy and Garcia-Pichel (2007) from its arid habitat, we tested the viability of previously desiccated *S. desiccabilis*, as shown in Figure 2. While viability was reduced to ∼1% over a week, viable cells were maintained over several more weeks, longer than tested for viability in brines.

### 3.2 Viability after brining

**FIG. 3.**
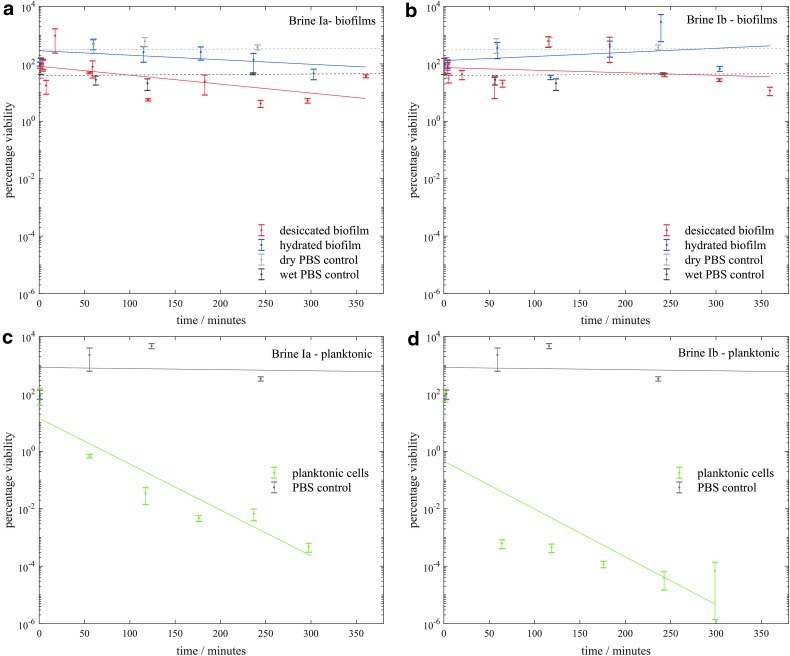
Viability of *S. desiccabilis* cells in desiccated and hydrated biofilms after periods of immersion in type I brines, measured by counting CFUs. **3a** and **3b** show biofilms in type Ia and Ib brines respectively and **3c** and **3d** show planktonic cells in type Ia and Ib brines respectively. Each data point is a biological triplicate result, with standard error bars, and trend lines are plotted as linear regressions of data from several systematic replicates. CFU, colony-forming unit.

**FIG. 4.**
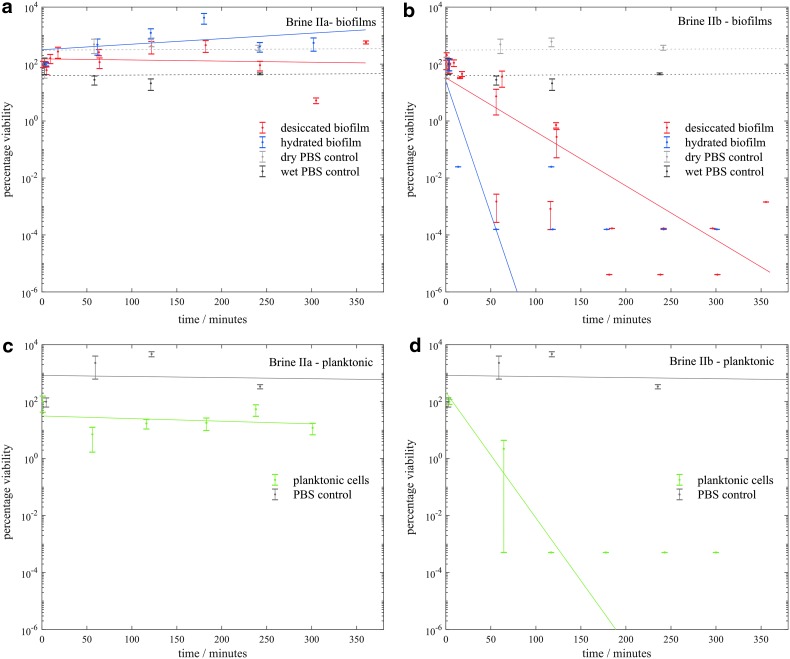
Viability of *S. desiccabilis* cells in desiccated and hydrated biofilms after periods of immersion in type II brines, measured by counting CFUs. **4a** and **4b** show biofilms in type IIa and IIb brines respectively and **4c** and **4d** show planktonic cells in type IIa and IIb brines respectively. Each data point is a biological triplicate result, with standard error bars, and trend lines are plotted as linear regressions of data from several systematic replicates.

**FIG. 5.**
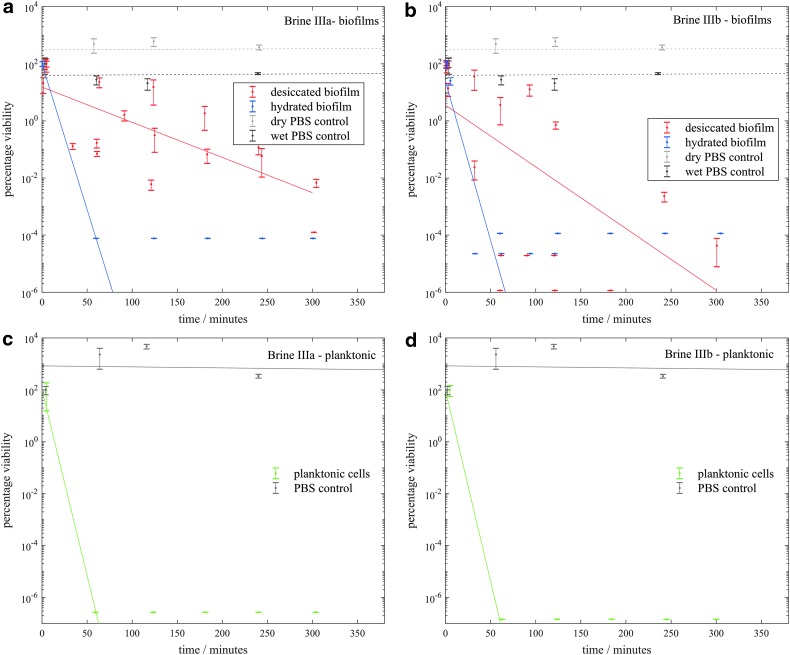
Viability of *S. desiccabilis* cells in desiccated and hydrated biofilms after periods of immersion in type III brines, measured by counting CFUs. **5a** and **5b** show biofilms in type IIIa and IIIb brines respectively and **5c** and **5d** show planktonic cells in type IIIa and IIIb brines respectively. Each data point is a biological triplicate result, with standard error bars, and trend lines are plotted as linear regressions of data from several systematic replicates.

**FIG. 6.**
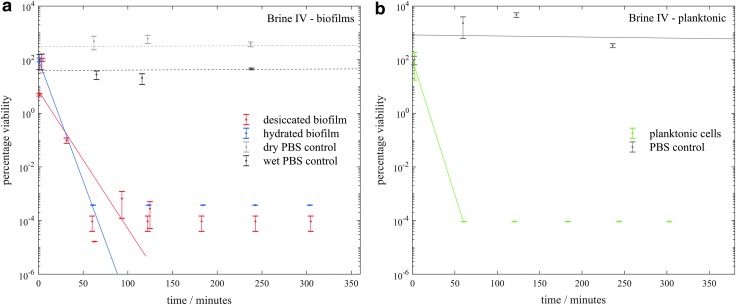
Viability of *S. desiccabilis* cells in desiccated and hydrated biofilms after periods of immersion in type IV brine, measured by counting CFUs. **6a** shows biofilms in type IV brine respectively and **6b** shows planktonic cells in type IV brine. Each data point is a biological triplicate result, with standard error bars, and trend lines are plotted as linear regressions of data from several systematic replicates.

Figures 3–6 show the viability of *S. desiccabilis* in planktonic and biofilm states over time when immersed in Mars-analog brines. Each time point was an independent culture (triplicate cell pellets or pieces of scoria with biofilm on them) exposed to brine for a given length of time. Experimental replicates are collected on the same axes. Error bars are the standard errors of the biological triplicates from each experiment. Best fit lines were calculated using a linear regression of all experimental replicates, not including time points after which the viability had reduced to zero. Controls performed in PBS instead of brines for both planktonic cell pellets and biofilms showed a viability that remained approximately constant over time, implying that loss of viability was due to the effect of the brines themselves.

In type I brines (Fig. 3) there were only minor decreases in viability over >300 min for biofilms when dried or when hydrated, but a general decrease in viability over >300 min for planktonic cells. For both hydrated and desiccated biofilms, there appeared to be some growth during immersion in brine Ib between 2 and 4 h.

In brine IIa (Fig. 4a, c), viability was maintained for over 300 min for planktonic cells, and dried or wet biofilms. In brine IIb (Fig. 4b, d) there was a sharp decline in viability in all three types of cell preparations, although the desiccated biofilms maintained viability for longer, up to between 2 and 3 h.

In type III brines (Fig. 5), viability of planktonic cells and cells in the hydrated biofilms reduced to below detection limits before the first measured timepoint, but the desiccated biofilms maintained viability in most cases to the 360 min timepoint. Brine IIIb has the lowest water activity, the lowest pH, and highest ionic strength of all the brines tested.

There was a rapid and complete loss of viability for both planktonic cells and hydrated biofilm cells in the type IV brine (Fig. 6), with the desiccated biofilm maintaining viability for longer, up to ∼2 h.

**FIG. 7.**
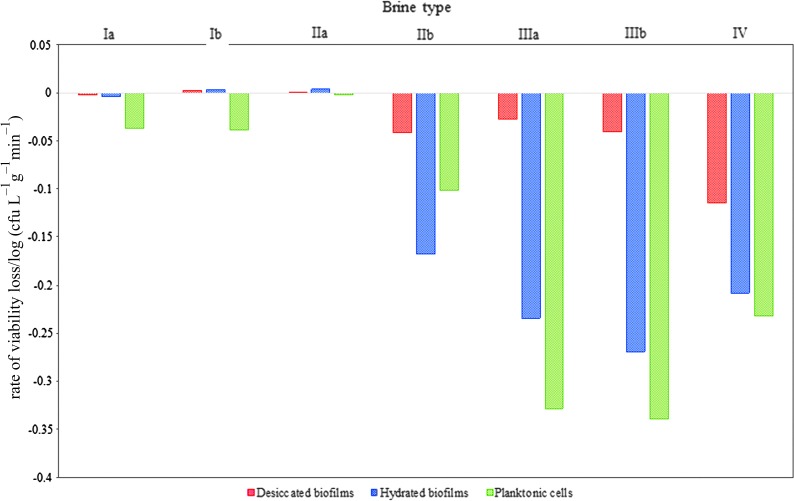
Rate of viability loss of *S. desiccabilis* in desiccated and hydrated biofilms and planktonic cells when immersed in brines Ia–IV, measured from linear regressions of the curves as shown in Figures 2–5.

To provide a more quantitative comparison of viability in the different brines, we calculated a linear regression for the results of each experiment and measured the gradient, as shown in Figures 3–6. Table 2 shows the gradient of these regression fits and the corresponding *R*^2^ value. A larger negative gradient implies a more rapid loss of viability. These values are graphed in Figure 7. Brines IIb, IIIa, IIIb, and IV show the most rapid loss of viability, and there is a clear difference between desiccated and hydrated biofilms, with the latter losing viability ∼2–10 orders of magnitude more rapidly when subjected to these brines.

**Table 2.** Rate of Viability Loss of *Sphingomonas desiccabilis* Cells in Desiccated and Hydrated Biofilms When Immersed in Brines Ia–IV, Measured from Linear Regressions of the Curves Shown in Figures 2–5

	*Rate of viability loss/log (CFU L^−*1*^ g^−*1*^ min)*				*Rate of viability loss/log (CFU L^−*1*^ min)*	
	*Desiccated biofilms*		*Hydrated biofilm*		*Planktonic*	
*Brine*	*Fit gradient*	*R*^2^	*Fit gradient*	*R*^2^	*Fit gradient*	*R*^2^
Ia	−1.84E-03	0.10	−3.63E-03	0.36	−3.68E-02	0.91
Ib	2.22E-03	0.09	3.27E-03	0.21	−3.83E-02	0.53
IIa	2.38E-04	0.08	4.45E-03	0.30	−2.11E-03	0.22
IIb	−4.11E-02	0.77	−1.68E-01	0.64	−1.02E-01	0.97
IIIa	−2.71E-02	0.50	−2.35E-01	1.00	−3.29E-01	1.00
IIIb	−4.07E-02	0.36	−2.70E-01	0.87	−3.39E-01	1.00
IV	−1.15E-01	0.71	−2.08E-01	1.00	−2.32E-01	1.00

CFU = colony-forming unit.

**FIG. 8.**
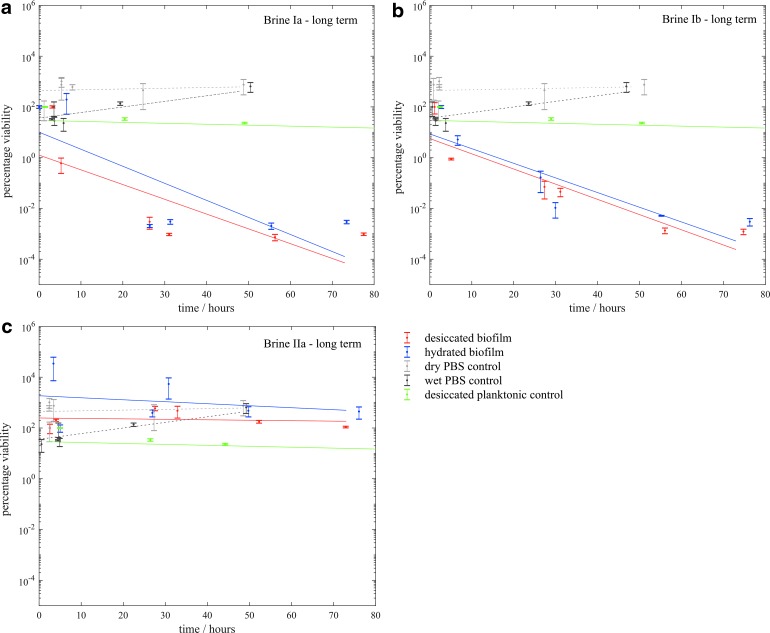
Viability of *S. desiccabilis* cells in desiccated and hydrated biofilms after periods of immersion in type Ia, Ib, and IIa brines over long timescales, measured by counting CFUs. Compared with PBS controls and long-term desiccation tests (Fig. 2). Where no CFUs were countable, results are plotted at a value of 1 CFU. Each data point is a biological triplicate result, with standard error bars, and trend lines are plotted as linear regressions of data from several systematic replicates.

For the brines where viability was not significantly affected over the course of several hours, we performed longer time series, as shown in Figure 8. Viability was observed to be reduced in both type I brine. Conversely, in brine IIa, viability was maintained over a period of several days.

### 3.3. Repeated brining cycles

We also tested the viability of biofilms on scoria after repeated cycles of brining and desiccation, repeating the process used in Section 2.1. Viability was tested after two to five cycles of brine immersion. CFU counts after the extraction procedure were negative for any more than a single cycle.

### 3.4. Biomass assay

**FIG. 9.**
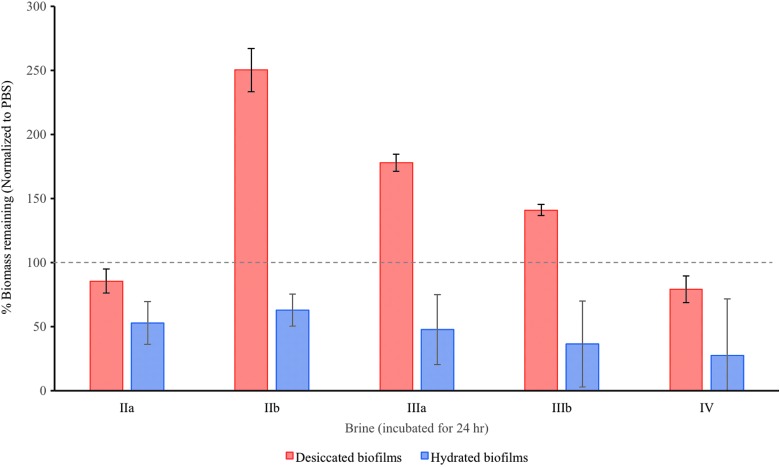
Percentage biomass remaining after immersion in brines IIa–IV for 24 h for desiccated and hydrated biofilms. Error bars are calculated from biological and systematic triplicates.

Given that the desiccated biofilms appeared to increase resistance to all the brines, they were investigated further. CV assays were used to investigate the quantity of EPS produced by *S. desiccabilis* in response to the simulated brines. As type I brines had little effect on the viability of cells in biofilms, these were not included in CV assays. Figure 9 shows the relative biomass present after a 24 h incubation in the types II, III, and IV brines for both desiccated and hydrated biofilms. All of the hydrated biofilms showed a reduction in biomass after 24 h, suggesting that the biofilms were broken down by the brines. In the case of the desiccated biofilms, there was also a reduction in biomass after 24 h in brines IIa and IV, but in brines IIb, IIIa, and IIIb the desiccated biofilms showed an increase in presumptive biomass over 24 h, by up to 250%.

**FIG. 10.**
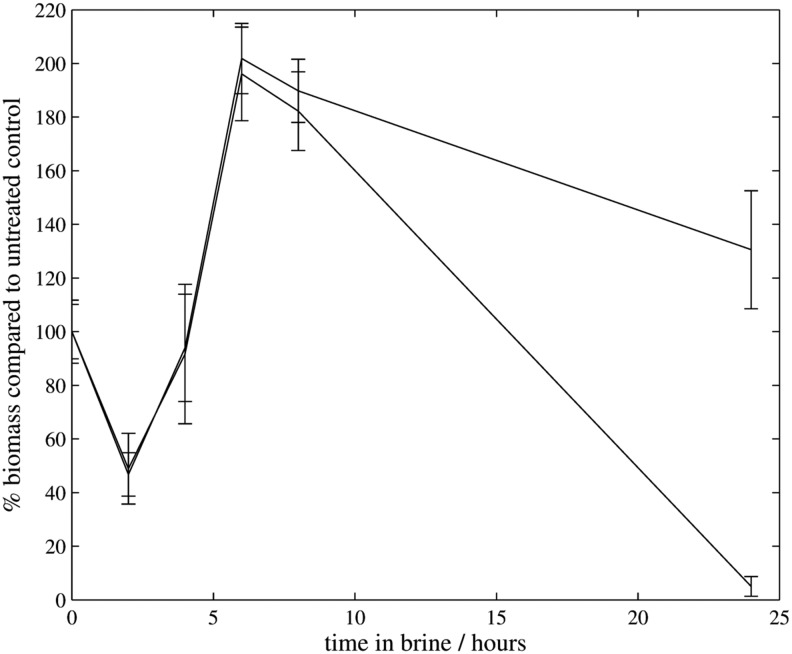
Biomass remaining over time for desiccated biofilms in brine IIb. Two experiments were performed.

To investigate this increase in biomass, we also performed a time series using the same experimental procedure, with brine IIb over another 24 h period (Fig. 10). During incubation in the brine there was an initial reduction in biomass followed by an increase over several hours, which peaked at ∼6 h after exposure to brine. After this increase, the biomass began to reduce from a peak of 200% to an average of ∼100% after 24 h.

## 4. Discussion

Other planetary bodies in the solar system harbor aqueous environments that differ markedly in geochemical composition from aqueous environments on Earth. These differences are a consequence of contrasting geological histories, which have produced the rocks and atmospheres that ultimately determine their water chemistries.

In this study, we sought to address the hypothesis that as on Earth, specific adaptations of microbial growth would influence the extent to which organisms could persist in extreme extraterrestrial aqueous environments. In particular, biofilms are known to provide resistance to chemical extremes (Luppens *et al.*, 2002; Zhang *et al.*, 2014). These data have implications for planetary protection, which is concerned with whether terrestrial micro-organisms could grow in extraterrestrial aqueous environments into which they are inadvertently introduced and, more speculatively, the adaptations that would be required for extraterrestrial life, if it exists, to grow in these alien geochemical extremes.

We found that the combined physicochemical extremes of the simulated martian brines had a significant effect on microbial survival. Generally, those brines with high water activity, low ionic strength, and moderate pH showed little immediate effect on the viability of *S. desiccabilis* cells, whether grown in a planktonic state or in a biofilm, although they were deleterious over longer timescales.

Brines with low pH and high ionic strength generally had a large impact on viability, making planktonic cells unrecoverable in <10 min. Our data suggest that water activity is one factor that influences the ability of biofilms to survive brine exposure. For example, brine IIIa has a high-water activity, low pH, and high ionic strength and had a lesser impact on viability than brine IIb, which has a similar pH and ionic strength but lower water activity. This observation is supported by the rapid loss of cell viability for planktonic cells and biofilms immersed in brine IIIb, which has the lowest water activity of the brines tested. That life is limited by water activity is well known (Stevenson *et al.*, 2014), but these data confirm that water activity plays a role in defining survival in a number of modeled martian brines.

However, factors other than water activity also appear to play a role in loss of viability in biofilms. Brine IV caused a more rapid loss in viability than brine IIb, although it has a slightly higher water activity, more moderate pH, and lower ionic strength. Brine IV is the only solution with perchlorate ions, which may suggest that these ions may be deleterious to cells. Brine IIa, which is rich in magnesium sulfate, appears to be the least deleterious brine of those tested, despite the fact that it has a lower water activity than brine Ia, and lower pH and higher ionic strength than both type I brines. This suggests that these physicochemical properties are not the only factor that controls the habitability of these brines, and that there are also ion-specific effects in their habitability. Other investigations have also suggested potential ion-specific effects, including some that promote survivability (Stan-Lotter *et al.*, 2002). These ion-specific effects could be due to particular chemical reactions generated in the presence of particular ions or due to microbes utilizing some of the brines' ions in their metabolism, making the response of micro-organisms to multi-ion environments difficult to predict. For example, perchlorate is generally highly oxidizing and biocidal (Wadsworth and Cockell, 2017), but some microbes are resistant and use perchlorate in metabolic pathways (Oren *et al.*, 2014).

The resistance of biofilms to loss of viability caused by the extreme brines is likely to be caused by a combination of physical protection and metabolic adaptation. Biofilms are known to provide a barrier between cells and external hostile environments (Monroe, 2007; Tseng *et al.*, 2013), in this case providing protection against the physicochemical extremes of the brine. As shown by the more rapid reduction in viability measured with hydrated biofilms compared with desiccated biofilms, the physical protection offered by the biofilm is not the only factor that allows the cells to remain viable for longer in the brine. Desiccation adds yet another level of protection against extreme brine exposure.

The CV assay suggested that when desiccated before brine exposure, biofilms showed an increase in presumptive biomass after several hours in the brine, a result not seen with hydrated biofilms and repeated consistently in desiccated biofilms. One biological explanation for these observations might be that desiccated biofilms are able to begin production of metabolites (such as membrane-stabilizing disaccharides) or additional EPS during initial exposure to matric stress. Production of EPS is a known response to osmotic stress/desiccation (Potts, 1994). Since matric (drying) water stress is equivalent to osmotic water stress from high concentration solutes (Potts, 1994), by drying the biofilms before brining, this process might act to prepare the organisms by signaling the production of osmotic-resistance metabolites (potentially changing the composition of the biofilm), which might continue during exposure to brine before loss of viability. These observations merit further study.

While our experiments show that under the most extreme brines, biofilms confer resistance to loss of viability, we were unable to recover viable cells after 5 h in the extreme brines (brines IIb, IIIa, IIIb, and IV). Furthermore, we tested the hypothesis that in the desiccated state, biofilms would enhance survival in transient aqueous systems, such as those proposed to occur in martian RSL. Under repeated cycles of desiccation and exposure to brine, which simulated the conditions that might occur in RSL, we found that viability was completely lost after the first cycle in all cases.

We have shown that biofilms formed by micro-organisms offer protection against the extreme conditions of Mars-analog brine solutions. Cells in biofilms desiccated before being immersed in brines remained viable for orders of magnitude longer than planktonic cells or hydrated biofilms under the same conditions. Some of these brines had low water activity, low pH, and high ionic content that would otherwise make them uninhabitable (Fox-Powell *et al.*, 2016), but *S. desiccabilis* biofilms retained viability in them for short periods of time, despite them quickly destroying *S. desiccabilis* cells in the planktonic state. Even though *S. desiccabilis* is not an extreme halophile, nor is it known to possess exceptional tolerance to osmotic shock, its biofilm-forming capability provides resistance to high levels of dissolved salts. It was selected here for this ability to form biofilms and known desiccation tolerance.

Our results add to the expanding body of literature showing the protective properties of biofilms in extreme environments. Previous studies have shown biofilms' capabilities to protect resistant cells against osmotic stress, but not against the unique combinations of physicochemical extremes exhibited by those used in our study, such as concentrated sulfate brines. These brines are particularly relevant to Mars, where perchlorate and sulfate ions are much more common than on Earth. Icy moons are also likely to have exotic combinations of ions that are unlike the chlorine-dominated terrestrial aqueous environments. However, there is some relevance to Earth, for example, locations such as the Basque lakes of British Columbia, which are sulfate-rich water bodies (Eugster and Hardie, 1978) or deep subsurface groundwater systems (Kietäväinen *et al.*, 2013). While biofilms may not offer the same levels of protection as sporulation in spore-forming organisms, they present a potential mechanism for maintenance of active biology in extreme nonhalite brine environments.

These results have implications for planetary protection (Rummel *et al.*, 2014). Although we cannot rule out longer term survival and growth of more extremophilic or extreme tolerant micro-organisms in these brines, our data show that an isolated micro-organism that naturally inhabits desert crusts and has tolerance to desiccation was rapidly killed in many of the brines, showing that some environments on present-day Mars that host liquid water are likely lethal to contaminants transferred to them on spacecraft or by human explorers. Nevertheless, we note that dilute brines analogous to early martian aqueous environments did allow for survival and growth of the nonextremophile species studied here, and that the relative survival was highly dependent on the different compositions of the brines and a combination of their physicochemical properties. Whether similar brines would act to destroy or even preserve biosignatures of any microbes they might have hosted is an open question and warrants further study.

## Abbreviations Used

CFUs: colony-forming units
CV: crystal violet
EPS: extracellular polymeric substances
RH: relative humidity
RSL: recurrent slope lineae
